# Structure-based chemical ontology improves chemometric prediction of antibacterial essential oils

**DOI:** 10.1038/s41598-024-65882-9

**Published:** 2024-07-01

**Authors:** Hiroaki Yabuuchi, Makiko Fujiwara, Akihiko Shigemoto, Kazuhito Hayashi, Yuhei Nomura, Mayumi Nakashima, Takeshi Ogusu, Megumi Mori, Shin-ichi Tokumoto, Kazuyuki Miyai

**Affiliations:** 1 Department of Pharmaceutical Industry, Industrial Technology Center of Wakayama Prefecture, Wakayama, Japan; 2 Department of Digital Manufacturing, Industrial Technology Center of Wakayama Prefecture, Wakayama, Japan; 3 Present Address: Kushimoto Branch, Shingu Health Center of Wakayama Prefecture, Wakayama, Japan; 4 Present Address: Tanabe Health Center of Wakayama Prefecture, Wakayama, Japan

**Keywords:** Machine learning, Antibacterial activity, Essential oil, Chemical ontology, Chemometrics, Cheminformatics, Data integration, Virtual drug screening

## Abstract

Plants are valuable resources for drug discovery as they produce diverse bioactive compounds. However, the chemical diversity makes it difficult to predict the biological activity of plant extracts via conventional chemometric methods. In this research, we propose a new computational model that integrates chemical composition data with structure-based chemical ontology. For a model validation, two training datasets were prepared from literature on antibacterial essential oils to classify active/inactive oils. Random forest classifiers constructed from the data showed improved prediction performance in both test datasets. Prior feature selection using hierarchical information criterion further improved the performance. Furthermore, an antibacterial assay using a standard strain of *Staphylococcus aureus* revealed that the classifier correctly predicted the activity of commercially available oils with an accuracy of 83% (= 10/12). The results of this study indicate that machine learning of chemical composition data integrated with chemical ontology can be a highly efficient approach for exploring bioactive plant extracts.

## Introduction

Plants are a great source of numerous bioactive compounds. Many researchers have isolated and identified potential phytochemicals from plants and developed new derivatives for medicinal purposes^1^. Additionally, these plants have been used as extracts that contain highly diverse compounds. Essential oils (EOs) are a type of plant extract obtained by distillation or expression, and are widely used in the pharmaceutical, agronomic, food, sanitary, cosmetic and perfume industries^2^. Although EOs typically exhibit milder antimicrobial activity compared with synthetic antibiotics, they have a great potential for overcoming antibiotic resistance, a growing problem in healthcare and livestock farming, through their multi-target effects of multiple constituents^3^.

Exploring novel medicinal plants is a major task in natural product research. The number of plant species in the world is estimated to reach 374,000^4^, but being mixtures of diverse compounds makes difficult to evaluate their efficacy and safety. Pharmacological studies have shown that the overall activity of the extracts cannot be described only by the presence of a few known constituents, but be a result of synergistic, additive, or antagonistic activity among a number of constituents^5^. Hence, an efficient methodology is needed for the medicinal plant exploration.

In the last decades, computational approaches have been developed to improve decision-making in drug discovery^6^ and in medicinal plant researches^7^. A discipline 'chemometrics' was proposed in 1970s and since then many chemometric applications have been reported to explore chemical data^8^. A chemometric model, called composition–activity relationships (CAR), was proposed to account for the relationships of the various chemical compositions of plant extracts with the bioactivity^9,10^. Recent studies using machine learning techniques have also shown good performance in predicting antibacterial^11,12^, antitumor^13^ and analgesic activity^14^ of the extracts. However, in practice, it is difficult to fit a multivariate model for them due to limited (seasonal, regional, ecological and legal) availability of the plant samples^1^. This limitation has hindered construction of a robust classifier for plant extracts containing hundreds of chemical constituents.

Chemical ontologies provide a standardised and hierarchical chemical classes for chemical compounds. Especially, recent development of structure-based chemical ontology, which provides structured classifications of chemical entities into hierarchically arranged chemical classes, has made it possible to automate annotation of numerous compounds^15,16^. However, to the best of the authors' knowledge, these ontologies have not been used for the CAR studies until now.

**Figure 1:**
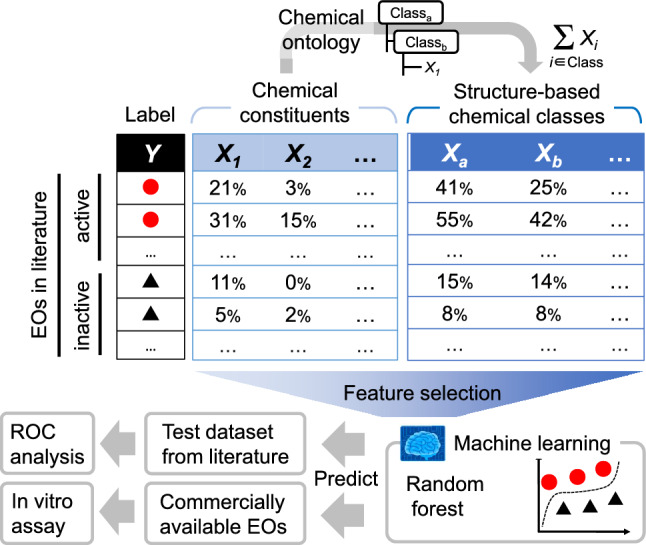
Overview of this study. The ratios of chemical constituents of that belong to each ontology class are summed to create a new compositional feature of essential oils (EOs).

In this study, we have shown that the structure-based chemical ontology has the potential to improve the active/inactive classification of antibacterial EOs. An overview of the study is shown in Fig. 1. The ratios of EO constituents that belong to each ontology class were summed up to create a new compositional feature. Feature selection was optionally performed to exclude irrelevant and redundant features and reduce the dimensionality. The all or selected features were trained to classify active/inactive EOs using a machine learning algorithm. We illustrate that the approach above improved the classification performance in two test datasets from literature and showed good performance in an antibacterial assay.

## Methods

### Data

A literature search on antibacterial EOs was performed using PubMed^17^ and Google Scholar^18^ in April 2021 and October 2023. The keywords “antimicrobial + AND + oil”, “antibacterial + AND + oil”, “bactericidal + AND + oil” and “microbicidal + AND + oil” were used for the search. The antibacterial test method was restricted to broth dilution, and the minimal inhibitory concentration (MIC) ≤ 1 mg/mL and > 1 mg/mL were interpreted as active and inactive EOs, respectively. The tested organisms were restricted to *Staphylococcus aureus* and *Escherichia coli*, the two most commonly studied bacteria for exploring the antibacterial activity of plant extracts^19^. The EOs from same plant species were removed to eliminate redundancy.

The chemical composition data of the antibacterial EOs were also retrieved through the literature search. Trace constituents with peak areas lower than 0.1% of the total ion chromatogram (TIC) were ignored. Chemical ontology (ChemOnt ver. 2.1) classes corresponding to the EO constituents were obtained from ClassyFire web application^15^ by inputting their chemical structures acquired from PubChem database^20^. The hierarchical structure of ChemOnt was also obtained from the ClassyFire web site.

### Reagents

Acetone for gas chromatography was purchased from KISHIDA CHEMICAL Co., Ltd, Japan. Dimethyl sulfoxide (DMSO) and thymol (special grade, purity 100.0%) were purchased from FUJIFILM Wako Pure Chemical Corporation, Japan. A series of *n*-alkane standards (C_9_–C_40_) was purchased from GL Sciences Inc., Tokyo, Japan. Mueller–Hinton II broth was purchased from Becton, Dickinson and Company, USA. *S. aureus* (NBRC 12732) for antibacterial activity tests was obtained from the National Institute of Technology and Evaluation, Biological Resource Center (NBRC), Japan.

### Machine learning of chemical composition with chemical ontology

For each ontology class in ChemOnt, ratios of EO constituents that belong to the class were summed to create a new compositional feature. The EO samples were divided into a training dataset and a test dataset according to whether the paper was published before or after December 2020. The antibacterial labels and features described by chemical constituents with or without ChemOnt classes of the training dataset were subsequently learned by a random forest^21^ to classify the active/inactive EOs against *S. aureus* and *E. coli*, respectively. The number of features used for feature subsampling (mtry) and splitting rule (split-rule) on the random forest were tuned by tenfold cross-validation using R ‘caret’ package (ver. 6.0–93). Then, the labels of the test dataset were predicted by the classifier, and the output probabilities of the active/inactive classification were evaluated by a receiver operating characteristic (ROC) curve^22^. The classification performance was also evaluated by precision, recall and F1 score. The training and prediction steps were repeated 10 times, and paired two-tailed *t*-test was used to determine whether there was any difference in the area under the ROC curve (AUC) between the methods. To measure the performance for EOs predicted to be active with high output probability, partial AUCs were calculated using ‘pROC’ (ver. 1.18.0) R package.

### Feature selection

Feature selection was performed to identify a subset of features (chemical constituents and ChemOnt classes) that can optimally differentiate active and inactive EOs in the training dataset for *S. aureus*. In this study, hierarchical information criterion (HIC)^23^ was employed as a feature ranking method that exploits the structure of hierarchical features. The HIC was originally developed to rank features with the number of patients in two groups. To apply the HIC algorithm to our data, we modified it as follows: (1) Mutual information estimator^24^ was introduced to calculate mutual information between continuous (chemical composition) and discrete (activity label) data. The estimator uses nearest-neighbor method to avoid problems with binning continuous data. (2) Branch statistical significance (comparing each feature in a branch to every other feature in the same branch) and tree statistical significance (comparing each feature to every other feature in the hierarchy) were determined by pairwise *t*-test instead of 2-proportion* z*-test. Although the exact weights of the branch and tree statistical significances can be calculated using frequency of non-zero values, they were set to 0.5 for model simplification in this study. The modified algorithm is shown in Algorithm 1.

**Figure:**
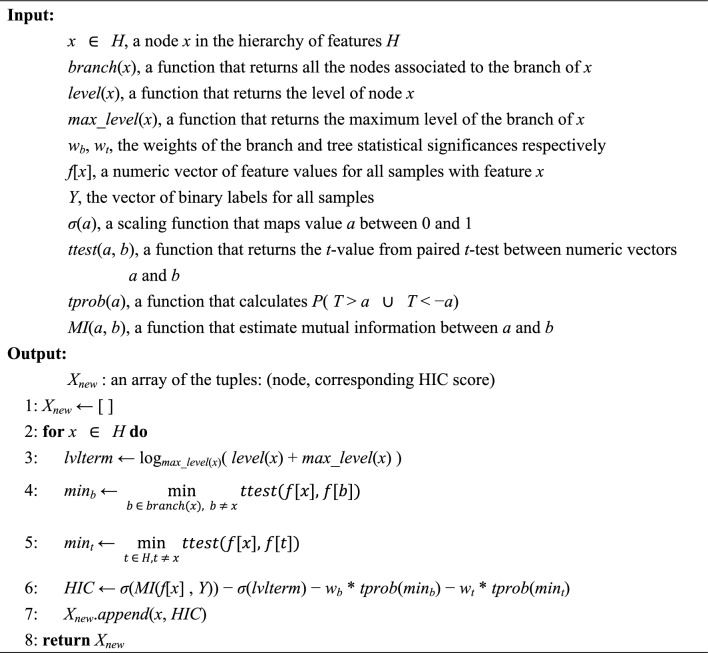
Algorithm 1. Hierarchical information criterion (HIC) ranking for continuous dataset.

An ablation study was conducted to investigate how each component of HIC (line 6 of Algorithm 1) influences the AUC. Mutual information and each of the other terms (hierarchical level, branch statistical significance and tree statistical significance) of HIC were used to rank the features, and tenfold cross-validation within training dataset was performed using the top *K* features (where *K* is 2, 4, 8, 16, 32, 64, 128 or 256).

To evaluate the effect of feature selection on the prediction performance, random forest classifiers were constructed from the top *K* features ranked by the HIC score. For comparison, principal component analysis (PCA) followed by training with the first *K* principal components (PCs) was performed using the chemical composition data (without ChemOnt classes) to determine whether a simple dimension reduction based on the variance affects the classifier’s performance.

For discussion of compositional difference between the training and test dataset, the high-dimensional EO composition data were embedded into a two-dimensional map using *t*-distributed stochastic neighbor embedding (t-SNE)^25^. The embedding was calculated using ‘tsne’ (ver. 0.1–3.1) R package.

### Gas chromatography/mass spectrometry (GC/MS) analysis

EOs from *Daucus carota var. sativus* and *Santalum album* were purchased from suppliers in Japan. Chemical characterization was performed in the same manner as that reported by the authors^26^ using a gas chromatograph coupled with mass spectrometer model QP2010 (Shimadzu, Kyoto, Japan). The EOs were dissolved in acetone (2 μL/mL). This solution (1 μL) was injected in split mode (1:50 ratio) onto a DB-5MS column (30 m × 0.25 mm i.d. × 0.25 μm film thickness, Agilent, USA). The injection temperature was set at 270 °C. The oven temperature was started at 60 °C for 1 min after injection and then increased at 10 °C/min to 180 °C for 1 min, increased at 20 °C/min to 280 °C for 3 min followed by an increase at 20 °C/min to 325 °C, where the column was held for 20 min. Mass spectra were obtained in the range of 20–550 m/z. EO components were identified based on a search (National Institute of Standards and Technology, NIST 14), the calculation of retention indices relative to homologous series of *n*-alkanes, and a comparison of their mass spectra libraries with data from the mass spectra in the literature^27,28^.

### Model evaluation by in vitro antibacterial assay

The antibacterial activity of the 12 commercially available EOs was predicted using the classifier for *S. aureus* with the best AUC and chemical composition data obtained by GC/MS analysis. The EOs were classified as active if the output probability ≥ 0.5, otherwise classified as inactive. The EOs from *Daucus carota var. sativus* and *Santalum album* were tested using the broth microdilution assay in the same manner as that reported by the authors^26^. A stock solution of each EO (dissolved to a concentration of 40 mg/mL in DMSO) was diluted to 4 mg/mL by Mueller–Hinton II broth medium, followed by serial dilution by the medium to lower concentrations (2, 1, 0.5, 0.25, 0.125, 0.0625, 0.0313, 0.0156 and 0.0078 mg/mL). Thymol, a known antibacterial agent, was dissolved and diluted in the same way to ensure microbial susceptibility as a positive control. The oils were all tested in triplicate. *S. aureus* NBRC 12732 was inoculated onto normal agar plates, and cultured for 24 h at 35 ± 1 °C. The bacterial suspensions were diluted with saline to obtain a 0.5 McFarland turbidity equivalent (*ca.* 10^8^ colony forming units per mL (CFU/mL)), and further diluted 10 times with saline (*ca.* 10^7^ CFU/mL). Then, 0.1 mL of EO-containing medium and 5 μL inoculum were added to sterile microtiter plates. 10% (v/v) DMSO in the medium was used as a negative control to determine if the solvent exhibited any antibacterial effect. The microtiter plates were incubated for 18 to 24 h at 35 ± 1 °C. Based on the opacity and color change in each well, the lowest concentration capable of inhibiting the growth was determined as the MIC.

## Results

### Data collection

The literature search identified 562 (270 active and 292 inactive) EOs for *S. aureus* and 495 (173 active and 322 inactive) EOs for *E. coli* with chemical composition data (Supplementary Table S1 and S2). 1,329 chemical constituents belonging to 327 ChemOnt classes for *S. aureus* and 1,307 chemical constituents belonging to 336 ChemOnt classes for *E. coli* were reported to compose the EOs (Supplementary Table S3, S4, S5 and S6). Among them, 413 (215 active and 198 inactive) EOs for *S. aureus* and 360 (134 active and 226 inactive) EOs for *E. coli* were published before December 2020 (training dataset), and the other 149 (55 active and 94 inactive) EOs for *S. aureus* and 135 (39 active and 96 inactive) EOs for *E. coli* were published after that month (test dataset).

### Machine learning of chemical composition with chemical ontology

The binary classifier models for *S. aureus* and *E. coli* were successfully constructed from the training dataset using composition data of all chemical constituents with/without integration of ChemOnt classes.

Both models for *S. aureus* exhibited comparable classification performance during cross-validation within training dataset (AUC = 0.79 vs 0.78). However, prediction for the test dataset revealed that the model constructed with ChemOnt classes performed better in AUC (0.748 vs 0.671, *p* = 7.0 × 10^−9^), AUC_0.5_ (0.279 vs 0.209, *p* = 5.9 × 10^−8^), AUC_0.2_ (0.060 vs 0.024, *p* = 9.6 × 10^−12^), AUC_0.1_ (0.020 vs 0.005, *p* = 6.2 × 10^−9^), precision (0.552 vs 0.514, *p* = 5.3 × 10^−4^), recall (0.744 vs 0.669, *p* = 4.0 × 10^−3^) and F1 score (0.634 vs 0.581, *p* = 4.9 × 10^−4^) than did those constructed without ChemOnt classes (Table 1 and Supplementary Table S7).

**Table 1:** AUC and partial AUCs for prediction of two test datasets.

Test organism	Metric	Comp + ChemOnt	Comp
*Staphylococcus aureus*	AUC	**0.748 ± 0.006**	0.671 ± 0.010
	AUC_0.5_	**0.279 ± 0.004**	0.209 ± 0.012
	AUC_0.2_	**0.060 ± 0.002**	0.024 ± 0.002
	AUC_0.1_	**0.020 ± 0.002**	0.005 ± 0.001
*Escherichia coli*	AUC	**0.722 ± 0.009**	0.650 ± 0.008
	AUC_0.5_	**0.278 ± 0.011**	0.214 ± 0.011
	AUC_0.2_	**0.079 ± 0.006**	0.045 ± 0.007
	AUC_0.1_	**0.027 ± 0.004**	0.013 ± 0.004

Composition data of chemical constituents (Comp) with/without chemical ontology (ChemOnt) classes were trained to classify active/inactive EOs. Prior feature selections were not performed for both input data. Values are means ± SD of 10 iterations, and the significantly better results are highlighted in bold (paired *t*-test, *p* < 0.01). AUC: Area under the receiver operating characteristic curve.

Likewise, both models for *E. coli* exhibited comparable classification performance during cross-validation within training dataset (AUC = 0.75 vs 0.75). However, prediction for the test dataset revealed that the model constructed with ChemOnt classes performed better in AUC (0.722 vs 0.650, *p* = 7.5 × 10^−9^), AUC_0.5_ (0.278 vs 0.214, *p* = 1.5 × 10^−6^), AUC_0.2_ (0.079 vs 0.045, *p* = 2.7 × 10^−6^), AUC_0.1_ (0.027 vs 0.013, *p* = 2.2 × 10^−5^), precision (0.640 vs 0.526, *p* = 1.5 × 10^−5^), recall (0.310 vs 0.205, *p* = 9.4 × 10^−4^) and F1 score (0.417 vs 0.291, *p* = 7.3 × 10^−4^) than did those constructed without ChemOnt classes (Table 1 and Supplementary Table S7).

### Feature selection

The ablation study for HIC showed that hierarchical level and branch statistical significance partially improved the AUC, whereas tree statistical significance did not yield the better AUC (Supplementary Figure S1A). Therefore, we omitted the tree statistical significance in the following evaluation.

**Figure 2:**
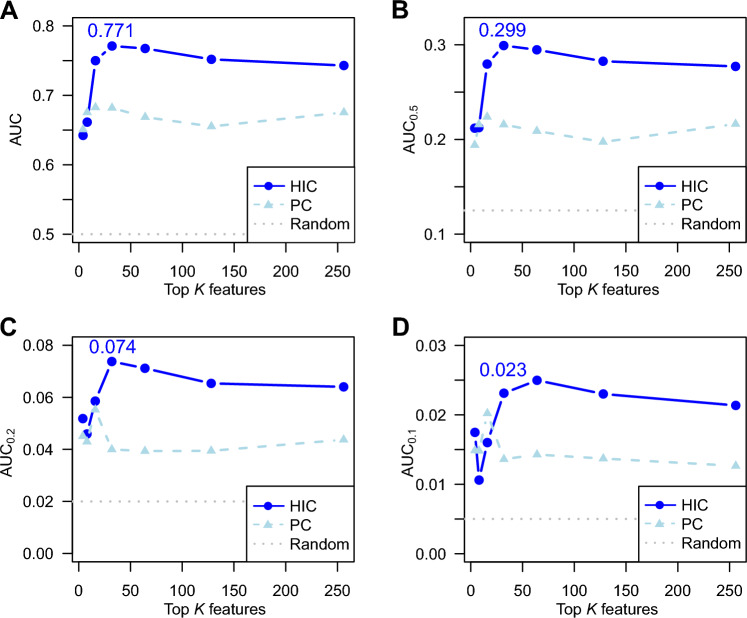
AUC and partial AUCs using the top *K* features of hierarchical information criterion (HIC). AUC (**A**), AUC_0.5_ (**B**), AUC_0.2_ (**C**) and AUC_0.1_ (**D**) vs the number of top *K* features for HIC are shown. For comparison, those of the first *K* principal components (PC) obtained by principal component analysis of chemical composition data (without ChemOnt classes) are plotted.

**Figure 3:**
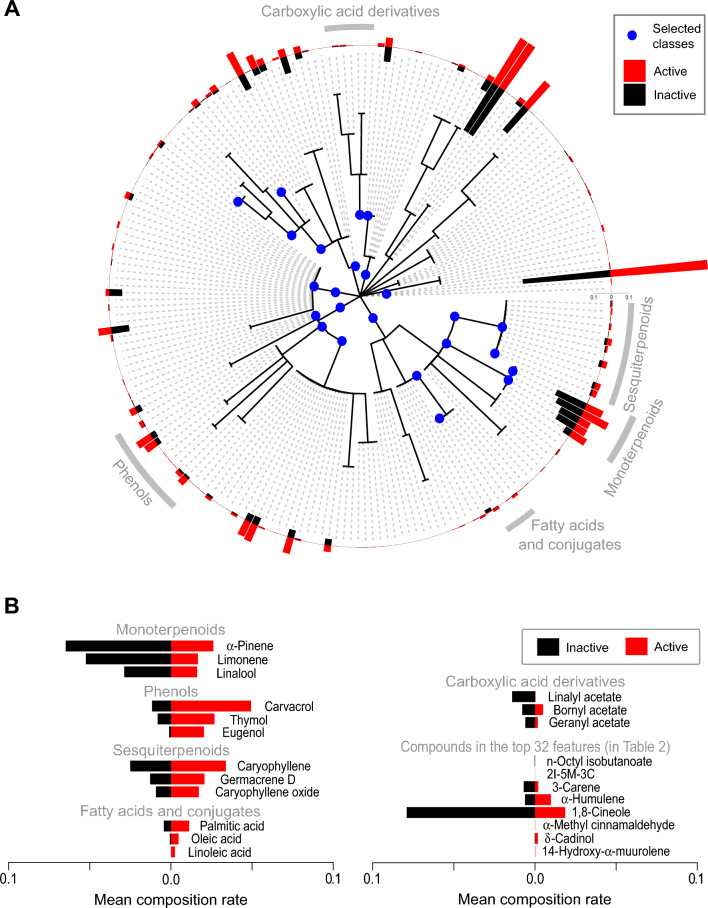
Chemical features selected by HIC in ChemOnt hierarchy. (**A**) The chemical classes in the 32 features are shown as blue circles. The positions of the classes mentioned in the Discussion section are shown in gray. The bar plot indicates the mean chemical composition of the active (red) and inactive (black) EOs. **(B)** Mean composition rate of the three most abundant constituents in the classes shown in Fig. 3A and those of chemical constituents in Table 2 are shown for active (red) and inactive (black) EOs. 2I-5M-3C: 2-Isopropyl-5-methyl-3-cyclohexen-1-one.

Feature selection by HIC revealed that the classifier using the top 32 features achieved the best AUC (0.771 ± 0.005), AUC_0.5_ (0.299 ± 0.009), AUC_0.2_ (0.074 ± 0.008) and F1 score (0.654 ± 0.009) (Fig. 2 and Supplementary Figure S1B–D). These values were greater than those performed using all features (Table 1 and Supplementary Table S7). The ChemOnt classes occupied 75% (= 24/32) of the selected features (Table 2). The hierarchical structure of the features is shown in Fig. 3 (and Supplementary Table S8) for data visualization. In contrast, the smaller AUC (0.683 of AUC at best) were observed using the PCs of chemical composition data (Fig. 2). The accumulated variance contributions of the first 16 and 32 PCs were 54% and 69%, respectively (Supplementary Figure S1E).

**Table 2:** The top 32 features selected by hierarchical information criterion.

Rank	ID	Class/compound name
1	**0001550**	**Sesquiterpenoids**
2	**0000333**	**Straight chain fatty acids**
3	**0000101**	**Eudesmane, isoeudesmane or cycloeudesmane sesquiterpenoids**
4	**0001401**	**Menthane monoterpenoids**
5	**0000012**	**Lipids and lipid-like molecules**
6	**0000262**	**Fatty acids and conjugates**
7	**0001549**	**Monoterpenoids**
8	**0000264**	**Organic acids and derivatives**
9	**0004325**	**Cyclohexenones**
10	**0001205**	**Carboxylic acids**
11	**0000134**	**Phenols**
12	**0002872**	**Elemane sesquiterpenoids**
13	**0002434**	**Alpha-hydrogen aldehydes**
14	**0002448**	**Benzenoids**
15	CID:61024	Octyl isobutyrate
16	**0000023**	**Naphthalenes**
17	**0001093**	**Carboxylic acid derivatives**
18	CID:573534	2-Isopropyl-5-methyl-3-cyclohexen-1-one
19	**0001564**	**Bicyclic monoterpenoids**
20	**0002279**	**Benzene and substituted derivatives**
21	**0001831**	**Carbonyl compounds**
22	**0003630**	**Organic 1,3-dipolar compounds**
23	CID:26049	3-Carene
24	**0000002**	**Organoheterocyclic compounds**
25	CID:5281520	α-Humulene
26	CID:2758	1,8-Cineole
27	**0000118**	**Ketones**
28	**0003940**	**Organic oxides**
29	CID:7557	α-Methyl cinnamaldehyde
30	**0000368**	**Dioxanes**
31	CID:3084311	Torreyol
32	CID:6428414	14-Hydroxy-α-muurolene

The chemical ontology (ChemOnt) classes are shown in bold.

**Figure 4:**
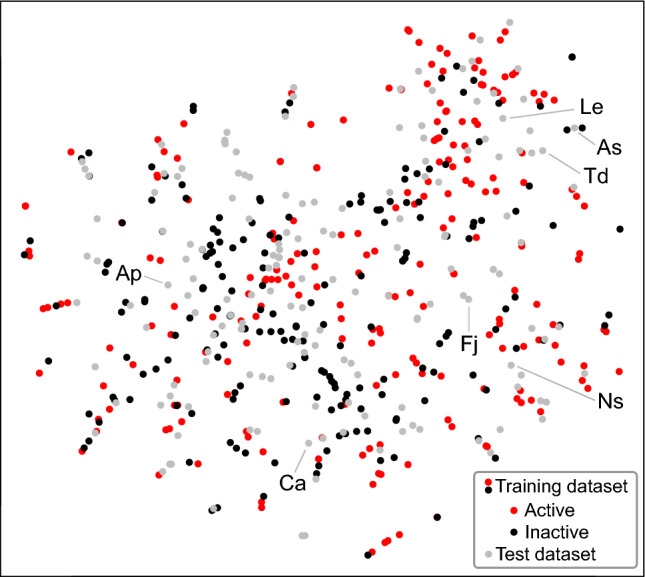
t-SNE visualization of the feature distribution of EO composition. The red and black dots indicate active and inactive EOs in the training dataset, respectively. Gray dots indicate EOs in the test dataset. Ap: *Acanthus polystachyus*, As: *Asarum splendens*, Ca: *Curcuma angustifolia*, Fj: *Farfugium japonicum*, Le: *Lannea egregia*, Ns: *Nepeta sessilifolia*, Td: *Thujopsis dolabrata*.

77 out of 149 EOs in the test dataset were predicted to be active by the classifier (Supplementary Table S9). *Nepeta sessilifolia* EO^29^ was correctly predicted to be active with the highest probability even though the main composition (oleic acid 62.1%, stearic acid 8.2%, linoleic acid 6.1%) was distinct from either of the trained EOs (Fig. 4). The classifier also correctly predicted other chemically distinct active EOs such as *Lannea egregia* (α-panasinsen 34.9%, β-caryophyllene 12.3%, α-copaene 11.4%), *Asarum splendens* (9-epi-β-caryophyllene 15.8%, eudesm-7(11)-en-4-ol 14.2%, β-caryophyllene 9.5%) and *Farfugium japonicum* (*cis*-3-hexen-1-ol 14.0%, *trans*-3-hexen-1-ol 13.7%, tetratetracontane 4.7%). In contrast, 12 active EOs (22% of total active EOs) were not correctively predicted by the classifier. They include EOs from *Acanthus polystachyus* (1-octadecanol 25.4%, *cis*-9-tetradecenoic acid isobutyl ester 23.0%, butyl 9-tetradecenoate 18.1%) and *Curcuma angustifolia* (curzerenone 25.3%, α-elemenone 13.6%, 1,8-cineole 11.6%).

### GC/MS analysis

The GC/MS analysis determined that the main constituents of *Santalum album* EO were valerianol (22.8%), 7-epi-α-eudesmol (11.6%), 10-epi-γ-eudesmol (9.3%) and elemol (8.5%), whereas those of *Daucus carota var. sativus* EO were carotol (38.3%), α-pinene (13.8%) and sabinene (7.9%) (Supplementary Table S10). The chemical profiles of the other 10 EOs (*Thymus vulgaris, Cinnamomum verum, Cymbopogon citratus, Origanum compactum, Trachyspermum ammi, Cedrelopsis grevei, Myroxylon balsamum var. pereirae, Leptospermum petersonii, Zingiber officinale* and *Thujopsis dolabrata*) were previously reported by the authors^26^. The major components of the 12 EOs determined by GC/MS analysis are summarized in Table 3.

**Table 3:** Chemical composition, predicted and observed antibacterial activity of commercially available essential oils.

Plant species	Major compounds identified (%) *	Probability	Activity (MIC) †	References
*Thymus vulgaris*	Thymol (60.7), *p*-Cymene (17.7), Carvacrol (5.2)	0.764	Active (0.5)	^26^
*Trachyspermum ammi*	Thymol (70.8), γ-Terpinene (15.5), *p*-Cymene (11.5)	0.758	Active (0.5)	^26^
*Origanum compactum*	Carvacrol (47.2), Thymol (16.2), γ-Terpinene (16.0)	0.728	Active (0.5)	^26^
*Santalum album*	Valerianol (22.8), 7-epi-α-Eudesmol (11.6), 10-epi-γ-Eudesmol (9.3), Elemol (8.5)	0.707	Active (1)	–
*Zingiber officinale*	α-Zingiberene (33.0), β-Sesquiphellandrene (13.8), *ar*-Curcumene (8.4)	0.685	Inactive (> 4)	^26^
*Thujopsis dolabrata*	Thujopsene (49.4), Cedrol (5.8), β-Bisabolene (4.2)	0.681	Active (0.5)	^26^
*Cedrelopsis grevei*	Ishwarane (31.6), β-Elemene (6.5), Guaia-6,9-diene (6.5)	0.669	Active (1)	^26^
*Daucus carota var. sativus*	Carotol (38.3), α-Pinene (13.8), Sabinene (7.9)	0.661	Inactive (2.5)	–
*Cinnamomum verum*	Cinnamaldehyde (59.1), Cinnamyl acetate (7.2), β-Caryophyllene (7.1)	0.655	Active (0.5)	^26^
*Leptospermum petersonii*	Geranial (38.5), Neral (31.9), Geraniol (8.4)	0.645	Active (1)	^26^
*Cymbopogon citratus*	Geranial (33.1), Neral (29.0), Citronellal (21.7)	0.532	Active (0.8)	^26^
*Myroxylon balsamum var. pereirae*	Benzyl benzoate (51.2), Benzyl cinnamate (32.2), Cinnamic acid (5.8)	0.483	Inactive (> 4)	^26^

*Values in parentheses are the percentage of the total peak area obtained from the total ion current chromatogram. † The values in parentheses are the minimal inhibitory concentrations (MICs in mg/mL) obtained by the antibacterial assay. The detailed chemical profile determined via GC/MS analysis is presented in Supplementary Table S10 and a previous report^26^.

### Model evaluation by in vitro antibacterial assay

The *Myroxylon balsamum var. pereirae* EO was predicted to be inactive, and the other 11 EOs were predicted to be active by the classifier constructed from the top 32 features. Antibacterial assay revealed that the MICs against *S. aureus* were 1 mg/mL for *Santalum album* EO and 2.5 mg/mL for *Daucus carota var. sativus* EO. The MICs of the other 10 EOs were already reported in a previous study^26^, and are also summarized in Table 3. The MIC for thymol (positive control) was 0.25 mg/mL, which was equivalent to literature data (0.03 v/v %^30^). No inhibition of bacterial growth was observed in the negative control.

In total, the classifier achieved accuracy of 83% (= 10/12) and AUC of 0.704 (Supplementary Figure S2) for the commercially available EOs. Among the EOs, *Thujopsis dolabrata* was correctly predicted to be active though the main components (thujopsene 49.4%, cedrol 5.8%, β-bisabolene 4.2%) were distinct from either of the trained EOs.

## Discussion

In this study, we collected the literature data of 522 antibacterial EOs composed of more than 1,300 compounds for machine learning classification. As expected from the high dimensionality of input data, the conventional chemometric classifier failed to show equivalent performance on the test dataset. The prior dimension reduction using PCA provided only a small improvement in the performance, probably because of high sparsity of the data and low accumulated variance contribution of the PCs. This result indicates that chemical diversity of antibacterial plant extracts is difficult to represent by low dimensional vectors from only chemical composition data.

A principle that compounds with similar structures (common structural features) possess similar biological activities is well accepted and has been applied to structure–activity relationship researches in medicinal chemistry^31^. Our approach incorporates the principle into composition–activity relationship by creating a higher-level feature set reflecting the similarity in molecular structures. Despite the small number of EO samples, the strategy constructed a robust classifier without significant overfitting in this study. Although the imbalanced (173 active vs 322 inactive) training dataset for *E. coli* caused low recall (0.2 to 0.3), the classifier retained good performance in ranking EOs ordered by the output probability (AUC = 0.72). Cost-sensitive learning or sampling technique may further improve the performance.

The 32 chemical classes ranked by HIC score (Table 2 and Fig. 3) provide insights in desirable chemical structures as an antibacterial agent. Phenols are a well-characterized class having antibacterial activity^32^. Carvacrol and thymol are the ones frequently found in antibacterial EOs, and have been used in dental applications and food flavorings^33^. Sesquiterpenoids, a structurally diverse class of C_15_ compounds composed of three isoprene units, were reported to show good antibacterial activity with MICs lower than 1 mg/mL^34^. Fatty acids are also well-known antibacterial agents in literature, and reported to show synergetic effect with several EO constituents^35^. In contrast, monoterpenoids (C_10_ compounds composed of two isoprene units) and carboxylic acid esters (included in “carboxylic acid derivatives” class) were reported to have antibacterial activity with higher MICs (> 1 mg/mL)^36^, which indicates that they were trained as inactive patterns. Another antibacterial assay using disc diffusion method also showed that ketones and esters (acetate esters) were less potent than corresponding alcohols among monoterpenes^37^.

Rare chemical constituents absent in the training dataset can influence the prediction performance. Our approach treats the constituents as a member of chemical classes if their chemical structures are identified. In this study, 158 rare constituents (observed not in training but in test dataset) were converted to either ChemOnt class, and utilized for prediction. However, 12% (on average) of the total composition was still unavailable in the literature (Supplementary Table S3). Development of an analytical technique and update of the mass spectral database will unveil the unknown composition of the EOs.

The development of the chemical ontologies and automated chemical classification systems has enabled us to easily represent a plant extract as a set of hierarchical chemical classes corresponding to a mixture of diverse compounds. Plant kingdom is estimated to contain between 200,000 and 1 million metabolites^38^. Recently, there is a growing number of papers devoted to antibacterial activity of the plant extracts^19^. The chemical ontologies will help us to predict their biological activity, and to understand potential core structures and functional groups of the metabolites via computational approaches.

Finally, the proposed method has potential limitations. The first is that the classification results depend on the hierarchy of chemical ontology. ChemOnt, the one used in this study, is based on core structures and functional groups. Theoretical model reflecting molecular descriptors may be a promising approach for reflecting molecular shapes and physical properties. The second limitation concerns the quantitativity. Regression analysis of MIC values is theoretically possible, but at present considered to be difficult because of the discrepancy in experimental conditions among studies. Large-scale bioassay data will support the evaluation of quantitative models.

The integration of chemical composition data with structure-based chemical ontology achieved better performance in predicting the antibacterial activity of EOs. Feature selection using hierarchical information criterion is also effective for avoiding overfitting and constructing an interpretable model. The results indicate that machine learning-based classification of the integrated chemical compositions data can be a highly efficient approach for exploring bioactive plant extracts.

### Supplementary Information

Supplementary Figures.

Supplementary Table S1.

Supplementary Table S2.

Supplementary Table S3.

Supplementary Table S4.

Supplementary Table S5.

Supplementary Table S6.

Supplementary Table S7.

Supplementary Table S8.

Supplementary Table S9.

Supplementary Table S10.

## Data Availability

Source codes and raw data are available at https://github.com/yabuuchi-hiroaki/chem-ont-predict-eo-activity. All other relevant data are within the paper and its Supplementary Information.
