# Supplementary material for: Structure-based chemical ontology improves chemometric prediction of antibacterial essential oils
Source: Sci Rep. 2024 Jul 1;14:15014. doi: 10.1038/s41598-024-65882-9 (PMC11217266; doi:10.1038/s41598-024-65882-9)
Supplement: Supplementary file 1 — Supplementary Figures. [file 41598_2024_65882_MOESM1_ESM.docx]

**Additional Information**

**Structure-based chemical ontology improves**

**chemometric prediction of antibacterial essential oils**

Hiroaki Yabuuchi^1*,#a^, Makiko Fujiwara^1^, Akihiko Shigemoto^2^, Kazuhito Hayashi^1,#b^, Yuhei Nomura^2^, Mayumi Nakashima^2^, Takeshi Ogusu^1^, Megumi Mori^1^, Shin-ichi Tokumoto^2^, Kazuyuki Miyai^1^

^1^ Department of Pharmaceutical Industry, Industrial Technology Center of Wakayama Prefecture, Wakayama, Japan

^2^ Department of Digital Manufacturing, Industrial Technology Center of Wakayama Prefecture, Wakayama, Japan

^#a^ Current Address: Kushimoto Branch, Shingu Health Center of Wakayama Prefecture, Wakayama, Japan

^#b^ Current Address: Tanabe Health Center of Wakayama Prefecture, Wakayama, Japan

* E-mail: yabuuchi_h0002@pref.wakayama.lg.jp (HY)

**Supplementary Information**

**Tables S1–S10**. (Microsoft Excel files)

**Figures S1 and S2**. (shown below)

**Figure S1. (A) AUC using the top *K* features of HIC with ablated terms.** Mutual information (MI) and each of the other terms (LV: hierarchical level, B: branch statistical significance, T: tree statistical significance) of hierarchical information criterion (HIC) were used to rank the features. **(B, C, D) Precision, recall and F1 score using the top *K* features of HIC. (E) Cumulative variance explained by principal components.**

**Figure S2. ROC curve for predicting antibacterial activity of commercially available EOs.** Composition data of chemical constituents (Comp) with/without chemical ontology (ChemOnt) classes were trained to classify the essential oils. Thirty two features (**Table 2**) were selected prior to training for Comp + ChemOnt data.
